# *In vitro* characterization of *Trypanosoma cruzi* infection dynamics in skeletal and cardiac myotubes models suggests a potential cell-to-cell transmission in mediating cardiac pathology

**DOI:** 10.1371/journal.pntd.0012288

**Published:** 2024-06-24

**Authors:** José María Eloy Contreras-Ortiz, Daniel Hernández-Mendoza, Claudia Márquez-Dueñas, Rebeca Manning-Cela, Moisés Santillán

**Affiliations:** 1 Centro de Investigación y de Estudios Avanzados del IPN, Unidad Monterrey, Apodaca, Nuevo Leon, México; 2 Departamento de Biomedicina Molecular, Centro de Investigación y de Estudios Avanzados del IPN, CDMX, Ciudad de México, México; 3 Centro de Investigación en Ciencias Biológicas Aplicadas, Universidad Autónoma del Estado de México, Toluca, México; US Food and Drug Administration, UNITED STATES

## Abstract

Chagas disease predominantly affects the heart, esophagus, and colon in its chronic phase. However, the precise infection mechanisms of the causal agent *Trypanosoma cruzi* in these tissue types remain incompletely understood. This study investigated *T*. *cruzi* infection dynamics in skeletal (SM) and cardiac myotubes (CM) differentiated from H9c2(2–1) myoblasts (control). SM and CM were generated using 1% fetal bovine serum (FBS) without or with retinoic acid, respectively. Initial invasion efficiencies and numbers of released parasites were equivalent between undifferentiated and differentiated cells (~0.3–0.6%). Concomitantly, parasite motility patterns were similar across cell lines. However, CM demonstrated significantly higher infection kinetics over time, reaching 13.26% infected cells versus 3.12% for SM and 3.70% for myoblasts at later stages. Cellular automata modeling suggested an enhanced role for cell-to-cell transmission in driving the heightened parasitism observed in CM. The increased late-stage susceptibility of CM, potentially mediated by cell-to-cell transfer mechanisms of the parasite, aligns with reported clinical tropism patterns. The myotube infection models provide novel insights into Chagas disease pathogenesis that are not fully attainable through *in vivo* examination alone. Expanding knowledge in this area could aid therapeutic development for this neglected illness.

## Author summary

*Trypanosoma cruzi* is a parasite that causes Chagas disease. While most initial infections are cleared by the immune system, some people develop a chronic infection that can emerge decades later and damage the heart or digestive system. This study aimed to understand why the heart is preferentially affected. We studied the infection dynamics of *T*. *cruzi* in different muscle cell models cultured in vitro: myoblast cells, and cardiac muscle-like or skeletal muscle-like cells differentiated from myoblasts. All three cell types were equally susceptible to initial parasite invasion. However, at later times, the cardiac muscle cell model displayed higher overall infection levels compared to the other cell types. Examining intracellular and released parasites over time revealed that cardiac cells harbored more parasites at later infection stages, despite similar parasite release across models. This ruled out shedding as the cause of disparate infection levels. Mathematical modeling suggested that higher rates of cell-to-cell transmission drive elevated late-stage infection in the cardiac model. Comparing infection kinetics between models provides insights into heart susceptibility to chronic damage from *T*. *cruzi* in vivo. Understanding disease mechanisms could aid prevention and treatment strategies for this important health issue.

## Introduction

*Trypanosoma cruzi* is a protozoan parasite that causes Chagas disease, a neglected tropical disease endemic to Latin America. This disease affects over 7 million people worldwide and causes over 12,000 deaths per year [1]. While most infections are asymptomatic, approximately 30% of chronically infected individuals will develop Chagasic cardiomyopathy or digestive megasyndromes later in life [2]. These pathologies are caused by the long-term parasite infection within heart and digestive tissue.

The mechanisms underlying preferential tissue damage in Chagas disease are not fully understood. Parasite-related factors such as invasion efficiency, intracellular growth kinetics, and motility are thought to play a role [3–5]. Host immune responses [6] and interactions between parasite and host cell signaling pathways also influence disease progression and pathology [7]. Understanding these disease mechanisms could help develop better prevention and treatment strategies.

*In vitro* cell culture models are a valuable tool for studying the infection dynamics of *T*. *cruzi*. Previous research in our laboratory studied parasite invasion and motility across diverse mammalian cell lines, finding that trypomastigotes detect and modify their movement according to cell type [5]. Our computational models also implicated the roles of host cell division rate and cell-to-cell transmission in shaping infection kinetics (4). Few studies have investigated *T*. *cruzi* infection in cardiomyocytes, obtained from embryonic [8] and neonatal mice [9], or skeletal [10] muscle cell cultures, that more closely mimic the relevant *in vivo* tissue targets. However, such investigations are significantly limited, as primary cells are highly fragile and have a very short viability window in culture conditions [11]. This poses a challenge for infection kinetics studies that demand long-term monitoring of the process.

This study aims to characterize aspects of *T*. *cruzi* infection using an *in vitro* model based on H9c2 myoblast differentiation into cardiac and skeletal myotubes. These cell types bear relevance as analogues of cardiac and skeletal myocytes [12–14]. H9c2 myoblast cultures offer an advantageous balance between sustained growth capabilities and preservation of certain biochemical and molecular phenotypes reflective of muscular tissues [11,14,15]. Through differentiation protocols, we generated myotube models to assess parasite invasion efficiency, characterize parasite motility in the presence of cells, study intracellular growth kinetics over time, and quantify released parasites in differentiated versus undifferentiated host cells.

Additionally, a mathematical modeling approach was utilized to simulate infection kinetics in the different cell types. A cellular automata model was developed incorporating parameters like parasite invasion, intracellular replication, and cell-to-cell transmission rates. By fitting the model to the *in vitro* infection data, we aimed to gain insights into the relative contributions of these factors in determining the infection dynamics observed experimentally in each cell type. Together, these analyses provide new insights into the cellular mechanisms underlying Chagasic cardiomyopathy versus skeletal muscle pathology.

## Materials and methods

### Cell culture and maintenance

NIH 3T3 embryonic mouse fibroblasts (ATCC CRL-1658) and H9c2(2–1) rat myoblasts (ATCC CRL-1446) were cultured in DMEM (Dulbecco’s Modified Eagle Medium [Gibco, ThermoFisher Scientific, USA]), supplemented with 10% fetal bovine serum (FBS) [Gibco, ThermoFisher Scientific, USA], 1% penicillin (10,000 units/mL), and 1% streptomycin (10,000 μg/mL) [Gibco, ThermoFisher Scientific, USA]. Cells were maintained at 37°C in a humidified atmosphere containing 5% CO_2_. H9c2(2–1) myoblasts were passaged when they reached approximately 70% confluence to preserve their differentiation potential. Prolonged culture at high confluence has been shown to impair the ability of H9c2(2–1) cells to differentiate into myotubes [5,11]. Therefore, myoblast stocks were maintained below 70% confluence through regular subculture to ensure retention of differentiation capacity for use in subsequent experiments.

### Parasite culture

Epimastigotes of the GFP-expressing CL Brener strain of *T*. *cruzi* [5] were cultured in LIT [Liver Infusion Tryptose] medium supplemented with 10% heat-inactivated fetal bovine serum (FBS), 1% penicillin (10000 u/ml)/streptomycin (10000 μg/ml), and 1% hemin (5 mg/ml) at 28°C [16]. To obtain infective trypomastigote forms, a protocol adapted from [17,18] was followed. First, a 60–70% confluent monolayer of NIH 3T3 fibroblasts was infected with 2×10^6^ mid-log-phase epimastigotes in DMEM with 2% FBS. After 48 hours, epimastigotes were removed by washing and the infected monolayer was maintained at 37°C in DMEM plus 2% FBS. Differentiating trypomastigotes were harvested from these cultures between days 7–12 post-infection. Only trypomastigotes released during this window were utilized for subsequent infection experiments.

### Differentiation of H9c2(2–1) myoblasts into skeletal and cardiac myotube models

H9c2(2–1) myoblasts at 60–70% confluence were trypsinized, collected by centrifugation at 612 g, and resuspended in DMEM with 10% FBS. Cell counts using a hemocytometer determined seeding densities of 600 cells/cm^2^ for myoblast controls or 6000 cells/cm^2^ for skeletal or cardiac myotube on 12 mm glass coverslips in 24-well plates. Cells were incubated at 37°C, 5% CO_2_ for 24 hours to attach. Medium was then replaced as follows: skeletal and cardiac myotubes received DMEM with 1% FBS. Additionally, cardiac myotubes received daily supplementation of 1 μM retinoic acid [Sigma Aldrich, USA] dissolved in DMSO [14] for 7 days, performed in the dark [11]. Myoblast controls remained in DMEM + 10% FBS. Media was changed every 3 days and morphological changes monitored daily for 7 days of differentiation. Coverslips were then fixed with 3.7% formaldehyde for 20 minutes, permeabilized with acetone at -20°C, and stained the actin cytoskeleton with rhodamine-phalloidin [Thermo Fisher Scientific, USA] 1:1000 dilution and nuclei with DAPI [Sigma Aldrich, USA] at 1:20000 dilution. Coverslips were mounted with Vectashield [Vector Laboratories Inc. USA] for analysis using a 40X fluorescence microscope. Actin cytoskeleton staining was used to delineate the contour of each cell and determine its length and area. This was done using the Straight line and Polygon tools in ImageJ (National Institutes of Health, Bethesda, MD) designed for this purpose. Cells from 10 random fields from three independent experiments, each conducted in triplicate, were evaluated. The number of nuclei per cell was determined using the LAS X software (Leica Microsystems CMS GmbH). The percentage of multinucleated cells was calculated relative to the total number of cells in 10 random fields from the three independent experiments, again each conducted in triplicate.

### Detection of troponin I3 (TNNI3) protein by Western blot

The presence of the cardiac muscle-specific protein TNNI3 was assessed in differentiated and undifferentiated H9c2(2–1) cells by Western blot. Myoblasts and cells undergoing skeletal or cardiac myotube differentiation were seeded in 60 mm dishes as previously described. After 7 days, cells were collected using trypsin-EDTA, washed twice with PBS by centrifugation at 612 g, and lysed by freezing in liquid nitrogen followed by thawing at 37°C in PBS containing protease inhibitors PMSF (1mM) [Sigma Aldrich, USA] and 1X Complete (Roche GmbH, DEU). Protein concentration was determined using Bradford assay against a BSA standard curve [19] in a 96-well plate reading at 595 nm [20]. 25 μg protein per sample was mixed with Laemmli buffer (Tris-Cl 60 mM pH 6.8, 2% SDS, 10% glycerol, 5% β-mercaptoethanol, 0.01% bromophenol blue), denatured by boiling, separated by 12% SDS-PAGE electrophoresis at 100 volts and 400 mA for 2 hours, and transferred to nitrocellulose membranes (Bio Rad, USA) in a Mini Trans-Blot (Bio Rad, USA) at 100 volts, 400 mA per hour. Transfer efficiency was verified with Ponceau staining. Membranes were blocked overnight in 6% Svelty milk-1X PBS at 4°C, then incubated with rabbit anti-TNNI3 antibody (1:500) [Abcam plc, USA] for 2 hours. Membranes were washed three times alternately with 1X PBS and Tween 20 (0.05%) 1X PBS and then incubated with a mouse anti-rabbit IgG-HRP secondary antibody [1:1000] (Santa Cruz Biotechnology Inc. USA) for 1 hour. After washing as above ECL (Thermo Fisher Scientific, USA) detection was used. Mouse anti-GAPDH IgG1κ monoclonal antibody (1:1000) and its secondary goat anti-Mouse IgG (H+L) Cross-Adsorbed, HRP (1:3000) (Invitrogen G-21040) served as a loading control. Signals were captured using a ChemiDoc imager.

### Evaluation of invasion efficiency, percentage of infected cells, intracellular parasite load and number of released parasites, through the infection kinetics of *T*. *cruzi*

H9c2(2–1) myoblasts were seeded on 12 mm round coverslips at 6000 cels/cm2 in 24-well plate and were grown (control) or differentiated into skeletal and cardiac myotubes, as described above. Cells were then infected with 1.8×10^5^ CLBr-GFP trypomastigotes (MOI 3), which is the optimal condition established in previous work [5] or with approximately 16 times more parasites (MOI 50). After incubation for 2 hours in DMEM with 2% FBS the non-adherent parasites were removed by washing with fresh no supplemented medium. Control myoblasts were incubated in DMEM plus 10% FBS and skeletal and cardiac myotubes in DMEM with 2% FBS at 37°C, 5% CO_2_. Coverslips were recovered at different times as necessary for each case, fixed, permeabilized, DAPI stained and mounted with Vectashield as described above, and analyzed by fluorescence microscopy. At 18 hours post-infection, infected cells were counted in ten random fields to determine invasion efficiency as (infected cells/total cells) x 100. The infection kinetics were studied by recovering coverslips every 24 hours for 7 days. The number of infected cells was determined in 10 random fields and calculated as the percentage of infected cells over total cells. The intracellular parasite load was also determined daily for 7 days in ten random fields throughout the infection period. Extracellular parasites (trypomastigotes and amastigotes) in the supernatant were quantified on days 5, 6 and 7 post-infections. This was done by centrifuging the supernatant at 612 g for 10 minutes and resuspending the pellet in 100 μl of PBS. The parasites were then counted using a Neubauer chamber. The experiments were performed as three independent experimental repeats, each conducted in triplicate.

### *Trypanosoma cruzi* motility analysis

Motility of *T*. *cruzi* trypomastigotes was assessed in the presence of myoblasts, skeletal myotubes and cardiac myotubes using live-cell imaging. Each cell type was cultured separately on Petri dishes at a density of 3×10^5^ cells per dish for 12 hours at 37°C and 5% CO_2_. Medium was then replaced with DMEM containing 2% FBS and 9×10^5^ trypomastigotes. Fluorescent polymer microspheres (Fluoro-Max green-fluorescent polymer micro spheres, Cat.No. G500) were added to monitor fluid flow, excluding experiments where convection was detected. Samples were maintained at 37°C and 5% CO_2_ for 1 hour prior to video recording at 50 frames/second (640x480 pixels) using an inverted fluorescence microscope (Nikon ECLIPSE TE2000-U) with a 40X objective at a temperature of 37–38°C. Custom image analysis software introduced in [5] was used to extract parasite and microsphere trajectories from videos. Trajectories were used to compute average speed and mean squared displacement over time. Mean squared displacement was fit to a power law function at short time intervals, with the exponent indicating if motion was subdiffusive, diffusive or superdiffusive. Superdiffusive particle motion was considered as indicative of convection.

### Statistical analysis

All quantitative data are expressed as the mean ± standard error of the mean (SEM) based on three independent experiments performed in triplicate. Statistical analyses were conducted using GraphPad Prism 8. Statistical significance was determined using one-way analysis of variance (ANOVA) to compare differences between multiple groups, followed by post-hoc tests as appropriate. Differences with a p-value less than 0.05 were considered statistically significant.

### Mathematical model development

A cellular automaton model was developed to simulate *in vitro* cellular infection dynamics. The model represents the cell culture surface as a regular square lattice, with each compartment occupied by one cell or empty. Empty compartments are denoted by *n* = 0. Uninfected cells are represented by *n* = 1, and infected cells by *n*>1, where *N*_*i*_ = *n*−1 indicates the number of intracellular parasites. The number of extracellular free-swimming parasites is given by *N*_*f*_. The model progresses iteratively through the following steps:

1. Cell replication–Uninfected cells replicate into a randomly selected adjacent compartment with probability *P*_*r*_ per time step. Specifically, a random adjacent site is selected for each cell, and if a randomly generated number is less than *P*_*r*_, replication occurs by occupying that site only if it is vacant. This probabilistic replication occurs independently for each cell at each time step. By representing cell proliferation as a random, probability-driven process constrained by available space, the model can capture the logistic growth trend typically exhibited by proliferating cell populations.
2. Infection by free parasites–Uninfected host cells could become infected by free parasites with a probability proportional to the current number of extracellular parasites (*N*_*f*_) and an assigned infection rate (*P*_*f*_). Specifically, at each time step the probability of a susceptible cell becoming infected was calculated as *P*_*f*_×*N*_*f*_. If a randomly generated number is lower than this probability, the cell becomes infected and *N*_*f*_ is decrement by one to represent the parasite occupying the newly infected cell and no longer being extracellular.
3. Infection by cell contact–Uninfected host cells become infected via contact transmission from infected neighboring cells. This mechanism of infection involves the localized transfer of individual parasites directly from an infected host cell to surrounding uninfected host cells through immediate contact. This differs from invasion by free extracellular parasites, which can move independently and infect distal cells rather than just neighbors. The probability of this occurring is calculated as the cell-to-cell transmission rate (*P*_*c*_) multiplied by the total parasite load (*N*_*n*_) in the adjacent infected cells. *N*_*n*_ represents the summed number of parasites in the 4 or 8 grid sites immediately surrounding the uninfected cell, depending on the specified cell line. If a random number falls below this probability, the uninfected cell becomes newly infected, representing local spread of parasites between cells over time.
4. Intracellular parasite growth–The number of intracellular parasites (*N*_*i*_) within each infected host cell is modeled as increasing logistically over time. Specifically, *N*_*i*_ undergoes updating at each time step, governed by the following discrete logistic growth equation:
$$N_{i,t+1}=N_{i,t+}+L(N_{i,t},a,b),$$
  With
$$L(N_{i,t},a,b)=a\Delta tN_{i,t}\left(1-\frac{N_{i,t}}{b}\right).$$
  In the above equations *a* is the intrinsic replication rate, *b* is the carrying capacity, and Δ*t* is the discrete simulation time step. This way of modeling intracellular parasite growth mimics the balanced processes of parasite proliferation and inherent limitations within the host cellular environment.
5. Cell lysis–Infected host cells undergo lysis with a probability dependent on the current intracellular parasite load (*N*_*i*_). Specifically, the lysis probability (*P*_*l*_) increases sigmoidally according to a Hill function, where the parameters *K* and *m* determined the shape of the curve:
$$P_{l}(N_{i},K,m)=\frac{N_{i}^{m}}{N_{i}^{m}+K^{m}}.$$
  At each time step, an infected cell lyses if a randomly generated number is lower than *P*_*l*_. When lysis occurs, the contents of *N*_*i*_ are added to the count of extracellular parasites (*N*_*f*_). This stochastic modeling of host cell rupture and subsequent parasite dispersal emulates how trypomastigotes are shed from host cells during infection to continue infecting new hosts and drive transmission.
6. Medium renewal–To replicate the effects of periodic medium changes in cell culture experiments, the model included regular reductions in the number of extracellular parasites (*N*_*f*_). At predefined time intervals, *N*_*f*_ was decreased by a pre-set fraction (*v*). This fraction represents the portion of parasites removed from suspension and replaced with fresh medium.

The pseudocode for the formerly described algorithm is as follows.

       Initialize model parameters:

       *P*_*r*_ = cell replication probability

       *P*_*f*_ = free parasite infection rate

       *P*_*c*_ = cell-cell transmission rate

       *a*, *b* = intracellular parasite growth rate parameters

       *K*, *m* = cell lysis Hill function parameters

       *v*, *t*_*r*_ = medium renewal fraction, interval

       *N*_*f*_ = Number of free-swimming parasites

       Δ*t* = Discrete simulation time step

       Initialize lattice:

       Randomly select one half of the lattice sites and set *n* = 1. Set all other sites to empty (n = 0).

       While *t*<total time:

          For each uninfected cell (*n* = 1):

              Replication:

                     If random()<*P*_*r*_ and randomly selected adjacent site empty:

                            Occupy adjacent site (set *n* = 1)

              Free parasite infection:

                     If random()<*P*_f_*N_*f*_:

                         Set *n* = 2, decrement *N*_*f*_ by 1

              Contact infection:

                     *N*_*n*_ = summed parasites of neighbors

                     If random()<*P*_c_*N_*n*_:

                     Set *n* = 1

       For each infected cell (*n*>1):

              Intracellular growth:

              *N_i_* = *N_i_*+*L*(*N_i,t_, a,b*)

       Cell lysis:

              If random()<*P*_*l*_(*N*_*i*_, *K*, *m*):

                     Add *N*_*i*_ to *N*_*f*_ and empty lattice site (set *n* = 0)

       Medium renewal:

              If *t* mod *t*_*r*_ = 0:

              *N_f_* = N_f_*(1–*v*)

       Increment *t* by 1

### Parameter estimation

Some model parameters were estimated from experimental data, while others were treated as free parameters and estimated by fitting model predictions to dynamic infection experiments. For fitting, plausible ranges for each free parameter were divided into 20 equal intervals. All possible parameter combinations were tested by running 100 simulations per combination and averaging the results. An error function was computed as the sum of squared residuals between the averaged simulation and experimental data points. The parameter set minimizing this error was selected. The estimation process for each parameter is detailed below.

Replication Propensity (*P*_*r*_)–For undifferentiated myoblasts (MB), this parameter was estimated by growing the cell line experimentally, counting cells over time, and running proliferation-only simulations matching experimental conditions. The lattice dimensions were determined based on maximum observed cell counts. *P*_*r*_ was then optimized through simulation iterations until modeled MB growth curves closely fit experimental data. The resulting value was *P*_*r*_ = 0.056 /hour. For differentiated skeletal (SM) and cardiac (CM) myotubes, *P*_*r*_ was set to zero, reflecting the inability of these cell types to proliferate once induced to form myotubes in culture.

Lysis Function (*K*, *m*)–To parameterize the lysis Hill function, direct manual counts were performed to quantify the intracellular amastigote loads observed across random microscope fields for each cell line. From these empirical parasite counts per cell; probability density functions were calculated describing the distribution of intracellular burdens. Examining the maximum amastigote loads observed in skeletal myotubes (SM), the parameter *K* was set at half this value (*K* = 47) for all three cell lines to represent the typical level of intracellular replication capacity reached prior to lysis. The exponent *m*, which controls the steepness of the lysis probability curve, was fixed at 10 based on sensitivity analysis. This produced an abrupt transition in the simulated probability of bursting that aligned with biological expectations.

Intracellular Carrying Capacity (*b*)–This parameter was defined as twice the lysis initiation threshold *K*.

Cell-to-cell transmission neighborhood–To represent the variation in inter-cellular contact between cell types in culture, different neighborhoods were defined for cell-to-cell parasite transmission. For undifferentiated myoblasts (MB), which grow in a dense and interconnected fashion, an 8-compartment Moore neighborhood was used to simulate cell-to-cell transmission. In contrast, the elongated morphology and spatial organization of differentiated skeletal (SM) and cardiac (CM) myotubes limited their contact to fewer surrounding cells. Therefore, a 4-compartment von Neumann neighborhood was adopted for these cell lines.

Infection Rates (*P*_*f*_, *P*_*c*_) and Growth Rate (*a*)–The parameters governing extracellular infection (*P*_*f*_), cell-to-cell transmission (*P*_*c*_), and intracellular parasite replication (*a*) were estimated by fitting a computational model simulating complete infection kinetics to time-series experimental data from multiple cell lines. Specifically, *P*_*f*_, *P*_*c*_ and *a* were iteratively optimized to minimize deviations between simulation outputs and measured infection levels over time. The invasion rate parameter *P*_*f*_, which controls the rate of parasite entry, was constrained to be equal across all cell lines given their similar levels of measured invasion efficiency. Meanwhile, the intracellular replication rate parameter *a* was fitted to produce simulation-derived distributions of intracellular parasite burdens matching those observed experimentally.

Removed Parasite Fraction (*v*)–This parameter was set to 0.9 based on sensitivity analysis. The analysis showed that values of *v* as low as 0.7 did not meaningfully change the model outcomes or predictions.

## Results

### Differentiation of H9c2 myoblasts into cardiac and skeletal myotubes

**Fig 1:**
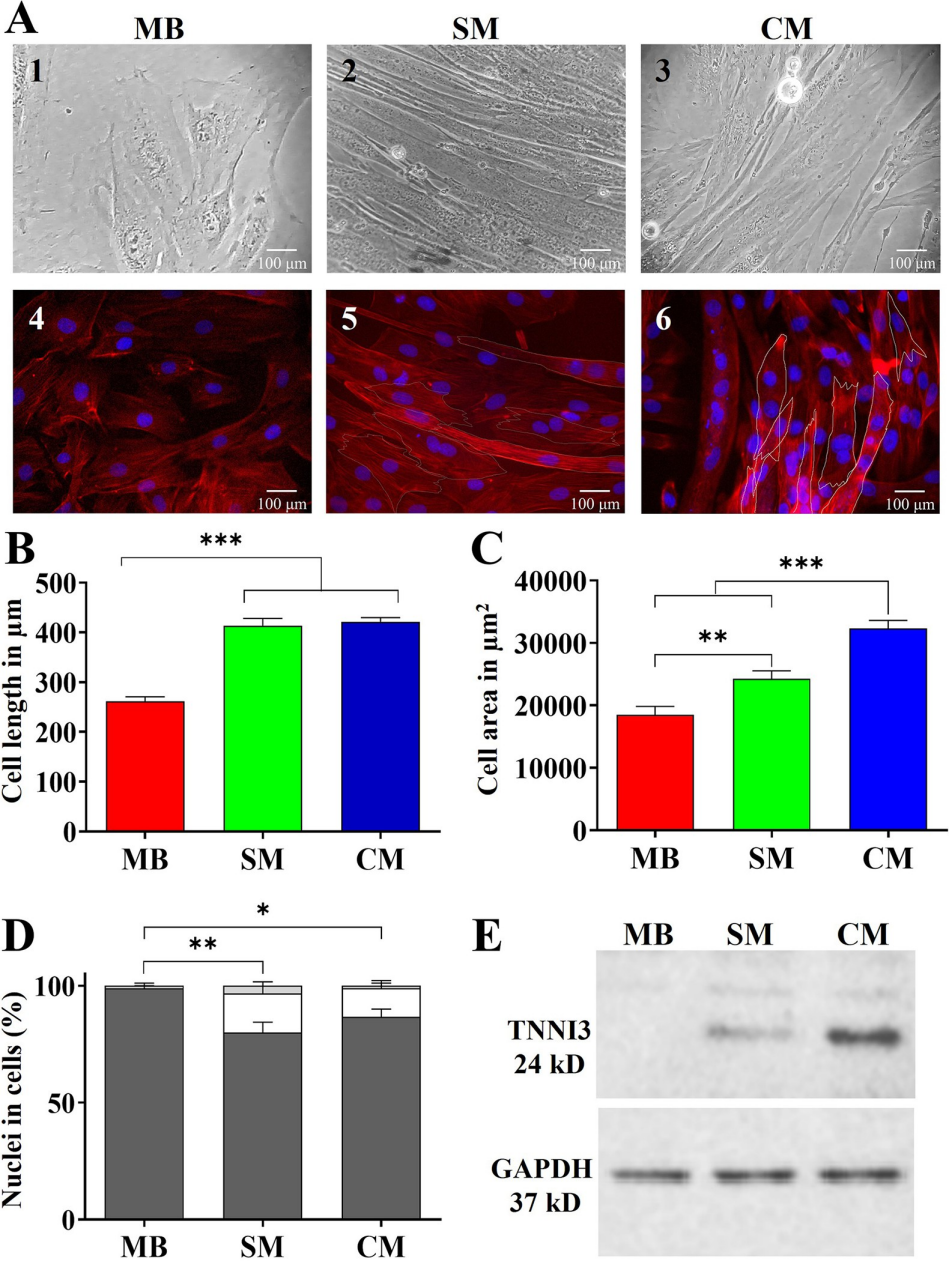
Morphological and molecular changes during myoblast differentiation. H9c2(2–1) myoblasts (MB) (panels A1, A4) were differentiated into skeletal myotubes (SM) by culturing in 1% FBS-DMEM (panels A2, A5) or cardiac myotubes (CM) by culturing in 1% FBS-DMEM with 1 μM retinoic acid (panels A3, A6) for 7 days. Panel A shows phase contrast (A1-3) and fluorescence microscopy with rhodamine-phalloidin/DAPI staining (A4-6). Scale bar = 100 μm. Panel B-D present length, area, and percentage of mono-nucleated (grey), bi-nucleated (White) and tri-nucleated (black) cells measured in MB, SM, CM from three independent experiments, each performed in triplicate. Data are mean ± SEM. Statistical analysis used ANOVA with Tukey’s test (***p<0.001, **p<0.01, *p<0.1 / 95.00% IQ of difference). Panel E shows a Western blot for TNNI3 and GAPDH loading control in total protein extracts of differentiated cell types.

We sought to differentiate H9c2(2–1) myoblasts according to established protocols to generate models of skeletal and cardiac myotubes (Fig 1) [10,11,13,14]. Both skeletal (panels A2 and A5) and cardiac myotubes (panels A3 and A6), exhibited elongated morphologies in all of their cells compared to the star-shaped appearance of undifferentiated myoblasts (panels A1 and A4). Additionally, the development of multinucleated cells was observed in the differentiated populations but not in predominantly mononucleated myoblast controls (panels A5 and A6). Quantification revealed significant increases in length (panel B), area (panel C) and multinucleation (Panel D) for both skeletal and cardiac myotubes versus myoblasts. Specifically, skeletal myotubes averaged 412.94 ± 45.75 μm in length, with an average area of 24260.4 ± 3891 μm^2^, while 20% contained multiple nuclei. Cardiac myotubes averaged 421.48 ± 25.46 μm in length, had the largest average area of 32344.7 μm^2^ ± 3769 μm^2^, and 13.34% were multinucleated. Myoblasts averaged 261.26 ± 28.42 μm in length and 18485.9 ^±^ 4072 μm^2^ in area, with only 1% exhibiting multinucleation.

Molecular analysis using western blotting showed higher expression of the muscle-specific marker troponin I3 in cardiac myotubes compared to skeletal myotubes (panel E). Myoblast controls exhibited negligible troponin I3 levels.

Taken together, the morphological and molecular alterations validate the successful differentiation of H9c2(2–1) myoblasts into models resembling skeletal and cardiac muscle lineages according to visual criteria and marker protein expression levels characteristic of the target tissue types.

### Invasion efficiency and motility patterns of *T*. *cruzi* in H9c2(2–1) myoblasts and differentiated skeletal and cardiac myotubes

**Fig 2:**
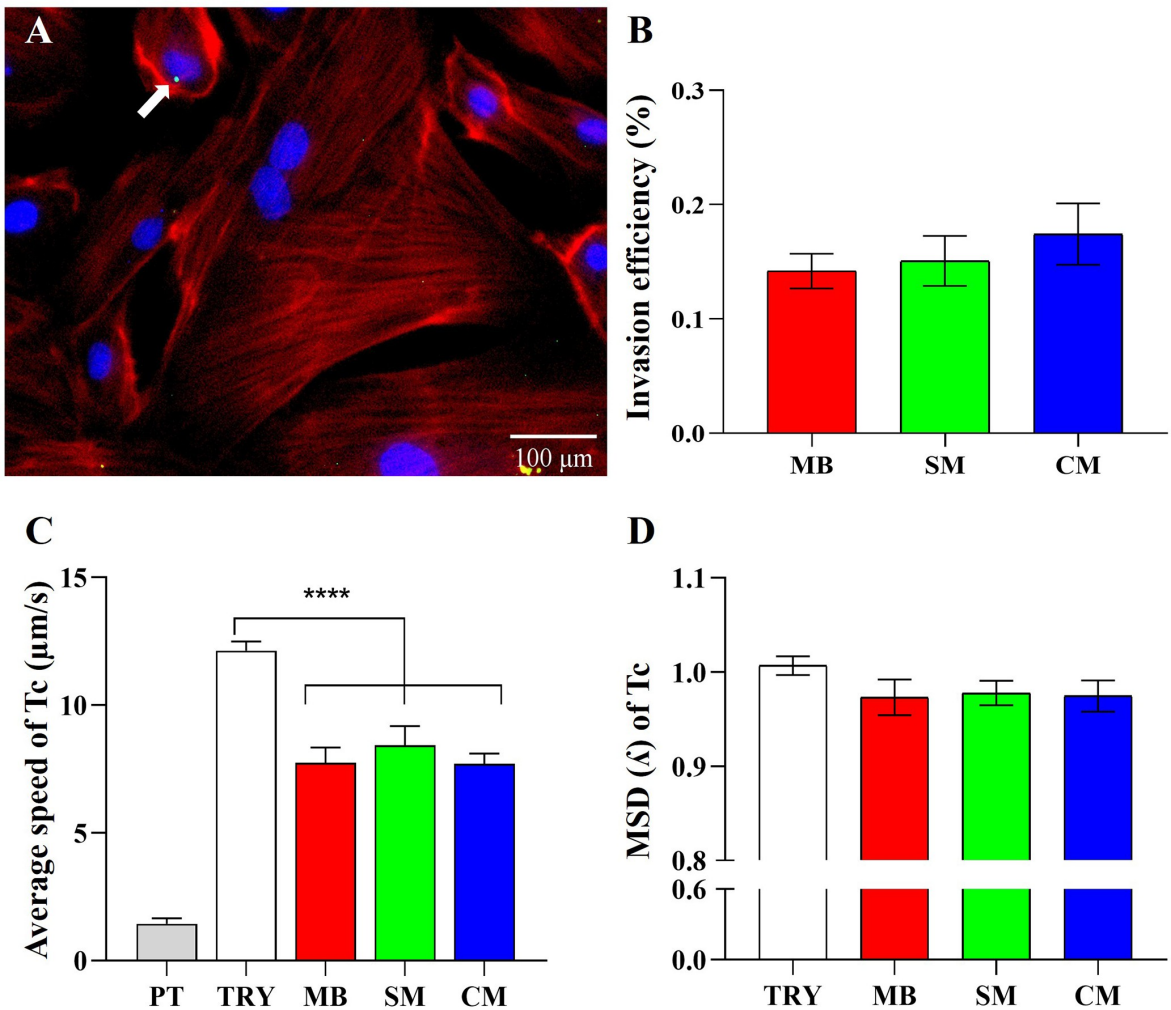
*T*. *cruzi* invasion and motility in myoblasts and differentiated cell models. (A) Fluorescence microscopy image of H9c2(2–1) myoblasts containing intracellular GFP-labeled amastigotes (green) and DAPI-stained nuclei (blue). Scale bar = 100 μm. (B) Invasion efficiency 18 hours post-infection, measured as percentage of cells containing intracellular parasites. (C-D) Motility analysis of *T*. *cruzi* trypomastigotes in myoblasts (MB), skeletal myotubes (SM), cardiac myotubes (CM). Controls included trypomastigotes alone (TRY) or fluorescent polymer microspheres exhibiting Brownian motion (PT). (C) Average parasite speed. (D) Mean squared displacement exponent (λ). Data are mean ± SEM from three independent experiments, each performed in triplicate. Panel B compared cell models by ANOVA where no significant differences were detected. Panels C-D compared groups by ANOVA with Tukey’s test (***p<0.0001 / 95.00% IQ difference). Motility was analyzed using parasite trajectories.

Following the successful differentiation of H9c2(2–1) myoblasts into skeletal and cardiac myotubes, we next evaluated *T*. *cruzi* invasion efficiency across cell types using a multiplicity of infection (MOI) of 1:3 (Fig 2). Myoblasts, skeletal myotubes, and cardiac myotubes grown on coverslips were infected with CLBr-GFP trypomastigotes. Invasion efficiency was determined 18 hours post-infection by quantifying the percentage of cells harboring one to three intracellular amastigotes (panel A), indicating trypomastigote differentiation into amastigotes and initiation of intracellular replication. No statistically significant differences were observed in invasion percentages between myoblasts (0.14%), skeletal myotubes (0.15%) and cardiac myotubes (0.17%) (panel B). S1 Fig also showed no statistically significant differences in invasion among MB, SM, and CM cells when a MOI of 1:50 was used. The percentage invasion at lower and higher MOIs followed the anticipated dose-dependent pattern, validating that invasion efficiencies do not vary between differentiated and undifferentiated H9c2(2–1) cell cultures. Based on these results, a MOI of 1:3 was used in subsequent experiments.

In a previous study, we reported that *T*. *cruzi* trypomastigote motility varies according to cell type and positively correlates with invasion efficiency [5]. Here, we evaluated average speed and mean quadratic displacement of trypomastigotes interacting with undifferentiated H9c2(2–1) myoblasts or differentiated skeletal and cardiac myotubes. Cell-free parasites and fluorescent microspheres exhibiting Brownian motion served as controls. As shown in Fig 2C, trypomastigote average speed significantly decreased among myoblasts (7.74 μm/s), skeletal myotubes (8.43 μm/s) and cardiac myotubes (7.70 μm/s) versus alone (12.12 μm/s). Regarding the mean quadratic displacement exponent (λ) (Fig 2D), a measure of initial position fluctuation, parasites alone exhibited near-diffusive motion (λ = 1.006). In contrast, among myoblasts (0.9730), skeletal myotubes (0.9775) and cardiac myotubes (0.9745), movement was subdiffusive (λ<1), without inter-line statistical differences. These results demonstrate that *T*. *cruzi* motility patterns change in the presence of cells, characterized by a decrement of average speed and a subdiffusive tendency to remain within localized areas, independent of host differentiation status. This aligns with our finding of unchanged invasion efficiencies across cell types. In sum, while trypomastigote movement varies near cells, this effect seems unrelated to host cells differentiation.

### Infection kinetics of *T*. *cruzi* in H9c2(2–1) myoblasts and differentiated skeletal and cardiac myotubes

**Fig 3:**
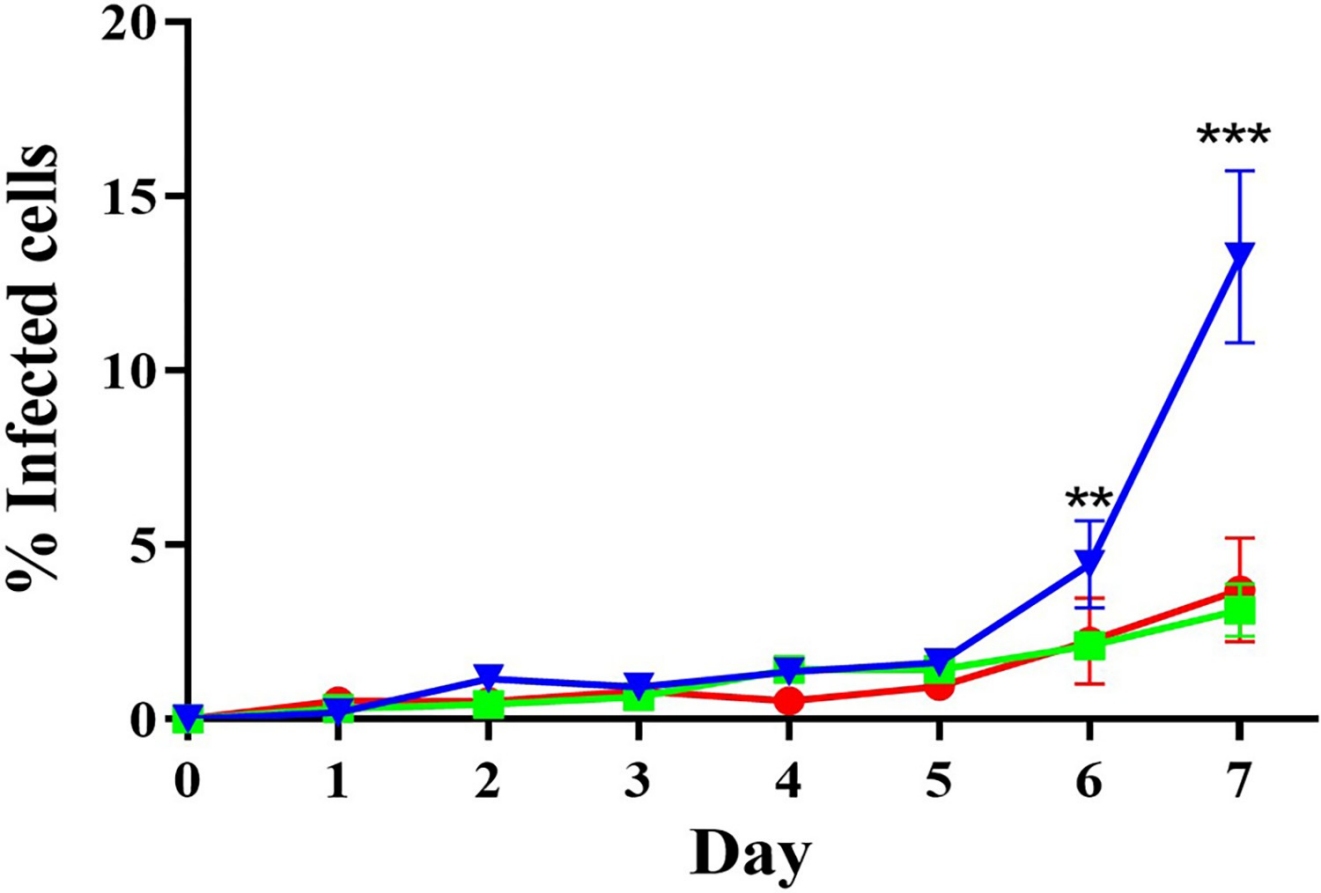
Kinetics of *T*. *cruzi* infection in myoblast and differentiated cell models. Line graph depicting percentage of infected cells in H9c2(2–1) myoblasts (red), skeletal myotubes (green) and cardiac myotubes (blue) over time. Data are mean ± SEM from three independent experiments performed in triplicate. Statistical analysis by ANOVA with Tukey’s test is indicated (**p<0.001 ***p<0.0001 / 95.00% IQ difference).

**Fig 4:**
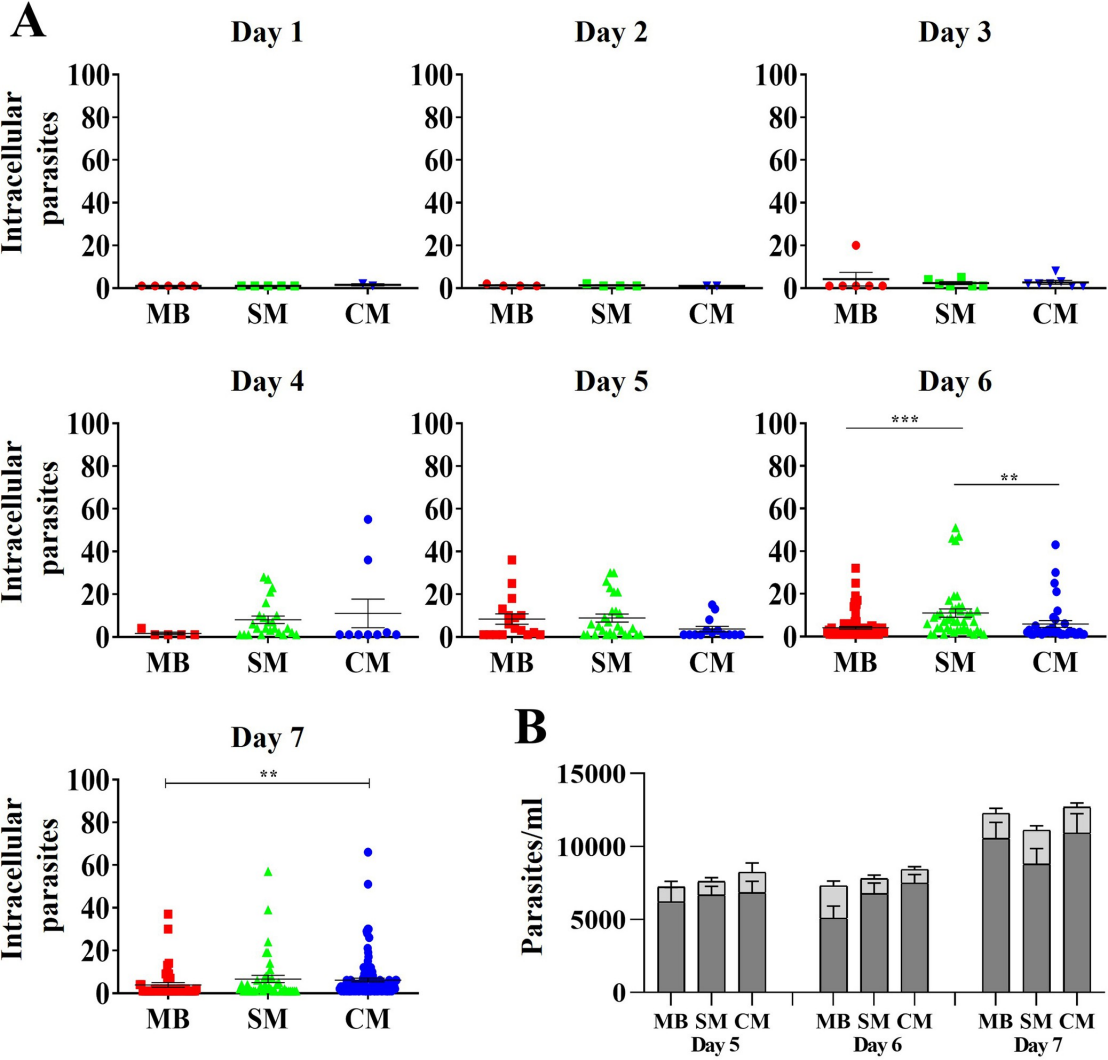
Kinetics of intracellular parasite load and release in myoblasts and differentiated cell models infected with *T*. *cruzi*. H9c2(2–1) myoblasts (MB) and skeletal (SM) and cardiac (CM) myotubes were infected with CLBr-GFP *T*. *cruzi* trypomastigotes. (A) Intracellular parasite number was quantified every 24 hours. (B) Number of trypomastigotes (light grey) and amastigotes (dark gray) released into the supernatant was measured at days 5, 6 and 7 post-infections. Data show mean ± SEM intracellular parasite numbers from three independent experiments performed in triplicate. Statistical analysis using Kruskal-Wallis with Dunn’s test was performed for panel A (*p<0.05, **p<0.01, ***p<0.0001/ 95.00% IQ of difference). No significant differences were detected by ANOVA for panel B.

Having established equivalent initial invasion efficiencies and similar motility parameter changes across cell types, we next examined post-invasion infection dynamics over time. Myoblasts, skeletal and cardiac myotubes on coverslips were infected with *T*. *cruzi* CLBr-GFP trypomastigotes and the percentage of infected cells quantified every 24 hours for 7 days. During the first 5 days, infection increased slightly and similarly in all cells (Fig 3A). However, by day 7 cardiac myotubes showed a significant rise to 13.26% infected cells compared to 3.15% for skeletal myotubes and 3.70% for myoblasts. We hypothesized that the larger cardiac myotube area (Fig 1C) could support higher parasite burdens and thus release more parasites when lysed. Intracellular (Fig 4A) and released parasite numbers (Fig 4B) were thus measured. On day 6, skeletal myotubes had significantly more intracellular parasites than myoblasts and cardiac myotubes. By day 7, cardiac myotubes supported higher numbers than myoblasts, yet supernatant release remained equivalent (Fig 4B). Therefore, the disproportionate cardiac myotube infection observed later cannot be attributed to differences in parasite release capacity.

### Role of potential cell-to-cell parasite transmission in H9c2(2–1) myoblast and differentiated skeletal and cardiac myotube infection dynamics

**Fig 5:**
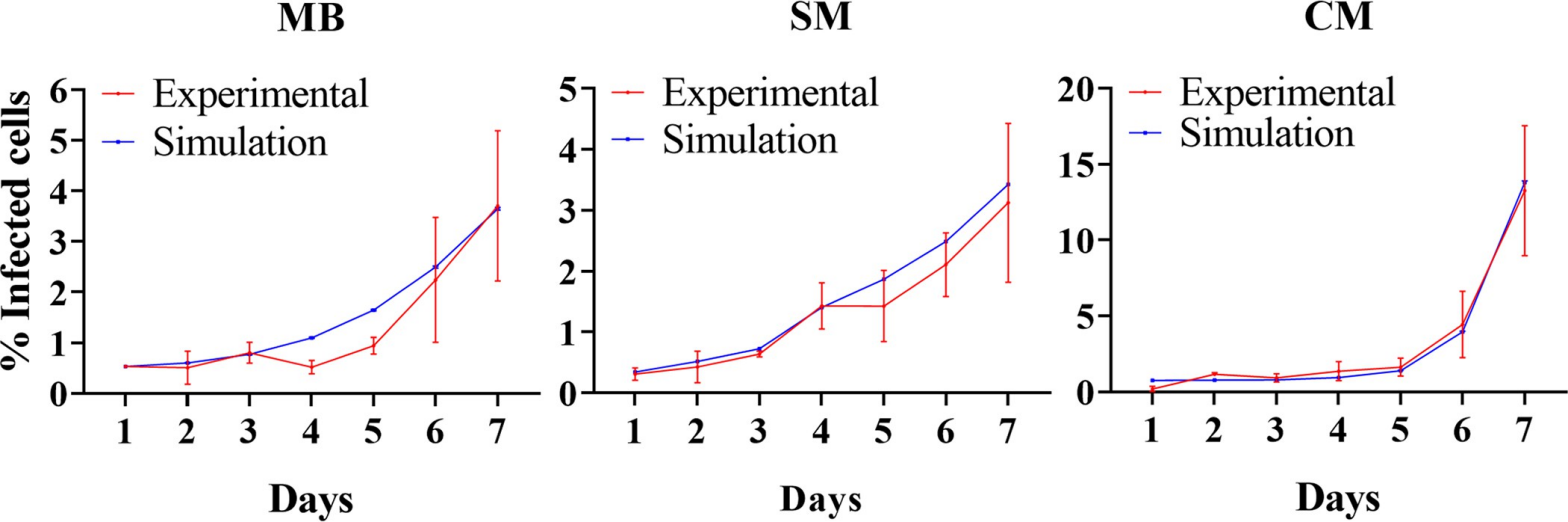
Mathematical model of *T*. *cruzi* infection kinetics in H9c2(2–1) myoblasts and differentiated cell models. Simulated (blue) versus experimental (red) infection kinetics data for H9c2(2–1) myoblasts (MB), skeletal myotubes (SM) and cardiac myotubes (CM). The cellular automata model accounted for parasite invasion, intracellular replication, cell division/death and cell-to-cell transmission. Best fits were obtained when using the same rate of infection by extracellular parasites across all cell lines. However, the reproduction and transmission rates were an order of magnitude higher in cardiac myotubes (CM) compared to other conditions, qualitatively reproducing the cardiac tropism observed experimentally.

A cellular automata modeling approach was undertaken to explore factors influencing the differential infection kinetics between cell types. A Model incorporating parameters for parasite invasion, intracellular replication, and potential cell-to-cell transmission was fitted to the experimental data (Fig 5). A discrete simulation time step Δ*t* = 1h was considered in the model. The free parasite invasion rate was determined by first simulating 18-hour infections under initial conditions matching those used experimentally, namely 90% confluency and a multiplicity of infection (MOI) of 3:1. Additionally, 90% of parasites were removed after the first 2 hours to reproduce parasite washing, again matching the experimental protocol. The invasion rate was then iteratively adjusted within the simulations until the predicted infection level after 18 hours averaged between cell lines aligned with the mean experimental value. The initial population of infected cells on day 1 was established as the value providing the best fit to the experimental data over the first 3 days for each cell line. The cell-to-cell transmission parameter was subsequently optimized to improve agreement between simulations and experiments at all timepoints. Finally, the intracellular parasite replication rate was adjusted to recapitulate the observed pattern of intracellular parasite burdens over time in the experimental data. Specifically, the estimated intracellular replication rates were 0.02/hour for myoblasts and 0.04/hour for skeletal and cardiac myotubes.

For myoblasts and skeletal myotubes, constant low cell-to-cell transmission rates of 5.5×10^−4^/intracellular parasite/hour and 1.4×10^−3^/intracellular parasite/hour achieved good fits, respectively. However, the cardiac myotube model required a time-dependent Hill function for this parameter, reaching 4.2×10^−2^/intracellular parasite/hour by day 7. The free parasite invasion rate was the same for all cell lines: 2×10^−10^/free parasite/hour. These adjustments qualitatively reproduced the escalating cardiac myotube infections over time. The simulations therefore support the hypothesis that elevated frequency of cell-to-cell transmission influenced the intracellular growth dynamics driving the preferential cardiac myotube parasitism observed experimentally at later infection stages. In the model, cell-to-cell transmission involved uninfected cells becoming infected via direct contact and localized parasite transfer between immediately neighboring infected cells, differing from invasion by freely motile extracellular parasites infecting distant targets.

## Discussion

In this study, we established that H9c2 myoblasts differentiated into skeletal and cardiac myotubes serve as a useful biological model for investigating the infective process and motility of *T*. *cruzi*. This provides insights into the infection dynamics in cell types resembling skeletal muscle and cardiomyocytes. We demonstrated morphological and molecular changes confirming successful differentiation of H9c2 cells into myotubes [11,21]. Cardiac myotubes displayed larger cell surface area compared to skeletal myotubes, as noted previously during myoblast differentiation [21].

In contrast to previous findings demonstrating variable *T*. *cruzi* invasion of different cell lines [5], the initial invasion efficiencies observed in this study were similar among undifferentiated and differentiated host cells. Concurrently, trypomastigote motility patterns were modified to a comparable extent in the presence of each cell type. However, cardiac myotubes showed significantly higher infection over time, suggesting increased susceptibility through secondary processes post-invasion. This agrees with the high parasitism observed in animal and human cardiac and skeletal muscle [22,23]. Variable invasion of embryonic and neonatal mouse cardiomyocytes was attributed to developmental differences in cell proteins [24] and parasite strains [9,25]. Our *in vitro*-derived myotubes may not fully express specific molecules conferring differential infectivity.

The increased late-stage infection of cardiac myotubes did not arise from higher intracellular parasite loads or release. This contrasts with trypanosomatid cell-to-cell spread via parasite-containing membranous extensions in macrophages and epithelial cells [26,27]. Our mathematical modelling suggested that cell-to-cell transmission plays a major role, increasingly activated over time in cardiac myotubes. *T*. *cruzi* induces plasma membrane disruption to hijack calcium-dependent membrane repair mechanisms and facilitate compensatory endocytosis of the parasite [28]. It is known that membrane injury causes ATP release, priming surrounding cells to swiftly repair new wounds via extracellular ATP signals [29]. As cardiac cells naturally maintain higher ATP [30] than skeletal cells and likely than myoblast could make them preferential long-term targets as the parasite leverages triggered repair pathways for repeated invasion cycle. Validating this will require future experimentation and a mathematical model to simulate the experimental outcomes. As intercellular dissemination facilitates immune evasion for intracellular pathogens, this mechanism may act synergistically with reported strain tropisms toward cardiomyocyte components to bias cardiac pathology, as described for other cytosolic bacteria and protists [31–34]. Strain-specific tropism of *T*. *cruzi* for cardiac myofibrils likely contributes to the observed kinetics [35,36]. Further work with TcI strains could better resolve differential myotube infectivity. Our integrated approach combining *in-vitro* and mathematical evidence provides mechanistic insight into factors influencing the tissue specificity of Chagas disease. Modulation of cell surface receptors or utilization of alternate parasite strains in this tractable myotube system could help clarify underlying determinants.

The mathematical model also showed a slight difference in the estimated intracellular replication rates, with myoblasts exhibiting a lower rate (0.02/hour) than skeletal and cardiac myotubes (0.04/hour). This observed discrepancy could be explained by the non-uniqueness of parameter fitting, as multiple combinations can minimize error when fitting against data. However, it is also plausible that metabolic changes occurring during differentiation enhanced intracellular parasite replication in skeletal and cardiac myotubes relative to myoblasts. Previous work has demonstrated that alterations in lipid metabolism promote higher *T*. *cruzi* proliferation within host cells [37]. Further investigation of metabolic changes induced in our myotube differentiation model would help confirm if they contributed to the higher fitted intracellular replication rates or if variability in parameter optimization was the more likely factor. Additional experimentation is needed to definitively distinguish between these possibilities.

## Conclusions

In this study, we successfully differentiated H9c2 myoblasts into skeletal- and cardiac-myotubes, establishing this as a biologically relevant *in vitro* model to study *T*. *cruzi* infection dynamics. The myotube lineages better resemble the *in vivo* targets of skeletal muscle and cardiomyocytes compared to undifferentiated myoblasts.

Initial *T*. *cruzi* invasion efficiency was equivalent amongst cell types. Similarly, trypomastigotes modified their motility patterns to a like extent in the presence of each cell type. However, infection kinetics differed significantly over time. Cardiac myotubes supported significantly higher infection levels by day 7 compared to skeletal myotubes or myoblasts. While the precise mechanism underlying this cardiac tropism requires further elucidation, our mathematical model suggested that parasite cell-to-cell transmission may play an important role in propagating infection within cardiac myotubes.

In conclusion, the use of H9c2-derived cardiac and skeletal myotubes, which more accurately mimic natural target tissues, can help expand current understanding of Chagas disease pathophysiology. This model presents opportunities to gain novel insights into host-parasite interactions and intracellular behavior governing cardiomyopathy outcomes. With optimization, it may allow exploration of how *T*. *cruzi* infection dynamics differ according to myocyte differentiation state. Overall, the cardiac selectivity observed underscores the value of this system for investigating Chagas pathogenesis.

## Supporting information

S1 Fig *T*. *cruzi* invasion in myoblasts and differentiated cell models. Invasion efficiency 18 hours post-infection with a multiplicity of infection (MOI) of 1:50, measured as percentage of cells containing intracellular parasites. Cell models were compared by ANOVA and no significant differences were detected.
(TIF)

S1 Data This zip file contains spreadsheets with the quantitative data used to generate the figures in the manuscript. The files allow reproduction of all graphs presented.
(ZIP)
