# Supplementary figures and images for: In vitro characterization of Trypanosoma cruzi infection dynamics in skeletal and cardiac myotubes models suggests a potential cell-to-cell transmission in mediating cardiac pathology
Source: PLoS Negl Trop Dis. 2024 Jun 24;18(6):e0012288. doi: 10.1371/journal.pntd.0012288 (PMC11226117; doi:10.1371/journal.pntd.0012288)

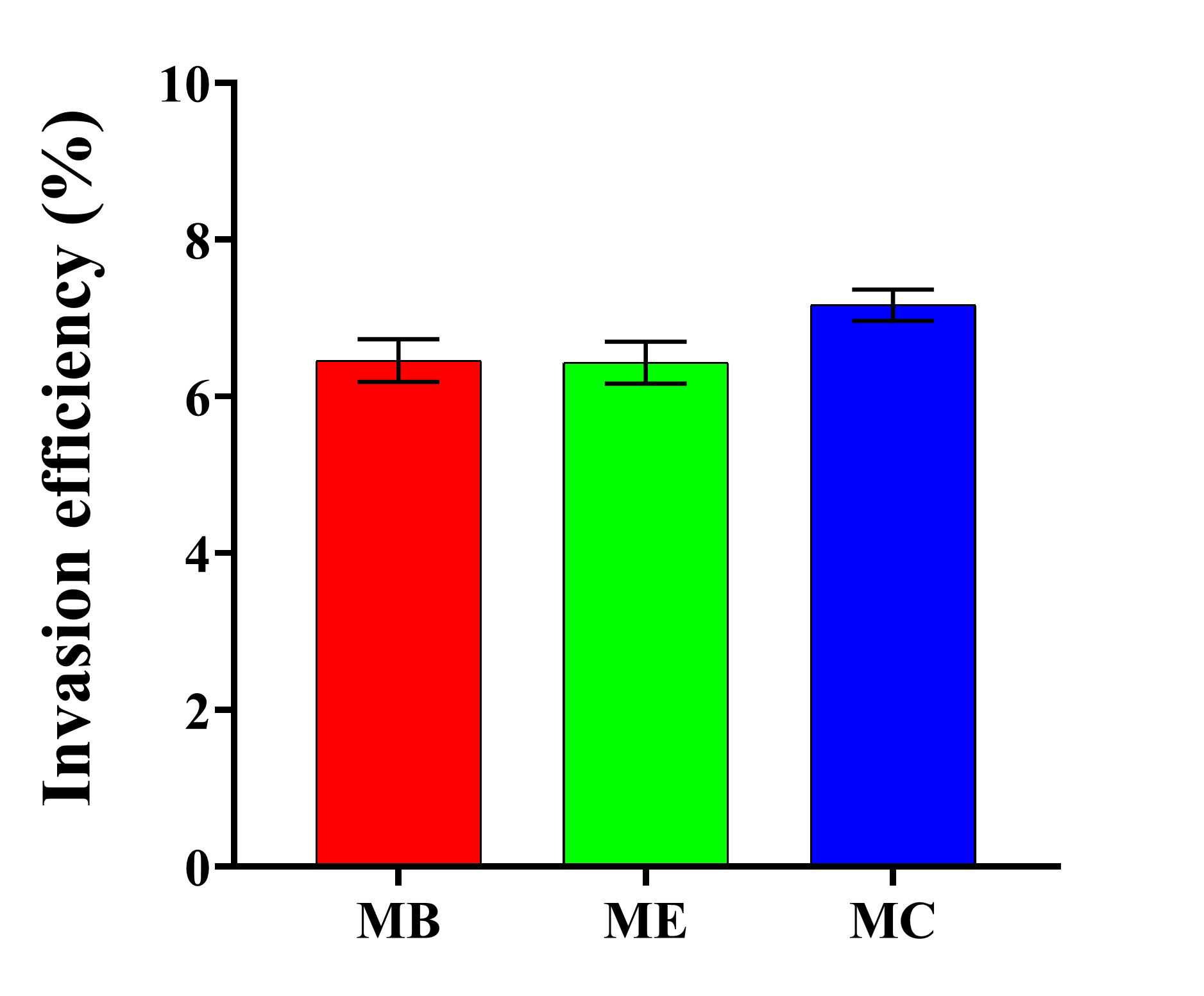

Supplement: S1 Fig — Invasion efficiency 18 hours post-infection with a multiplicity of infection (MOI) of 1:50, measured as percentage of cells containing intracellular parasites. Cell models were compared by ANOVA and no significant differences were detected. (TIF) [file pntd.0012288.s001.tif]
